# Expression of membrane fusion proteins in spermatozoa and total fertilisation failure during in vitro fertilisation

**DOI:** 10.1111/andr.13215

**Published:** 2022-07-01

**Authors:** Simona Ioana Enoiu, Marie Berg Nygaard, Mona Bungum, Søren Ziebe, Morten Rønn Petersen, Kristian Almstrup

**Affiliations:** ^1^ The Fertility Clinic Rigshospitalet University of Copenhagen Copenhagen Denmark; ^2^ Department of Growth and Reproduction Rigshospitalet University of Copenhagen Copenhagen Denmark; ^3^ Reproductive Medicine Centre Skåne University Hospital Malmo Sweden; ^4^ International Center for Research and Research Training in Endocrine Disruption of Male Reproduction and Child Health (EDMaRC) Rigshospitalet University of Copenhagen Copenhagen Denmark; ^5^ Department of Cellular and Molecular Medicine Faculty of Health and Medical Sciences University of Copenhagen Copenhagen Denmark

**Keywords:** fertilisation failure, IVF, membrane fusion, spermatozoa

## Abstract

**Background:**

Couples increasingly experience infertility and seek help from assisted reproductive techniques to become pregnant. However, 5%–15% of the couples that are selected for in vitro fertilisation (IVF) experience a total fertilisation failure (TFF), where no zygotes develop despite oocytes and semen parameters appear to be normal. We hypothesise that TFF during IVF could be related to improper membrane fusion of gametes.

**Objective:**

To investigate the membrane integrity and fusion proteins in spermatozoa from men in couples experiencing TFF.

**Materials and methods:**

A total of 33 infertile couples, 17 of which experienced TFF during IVF and 16 matched control couples with normal IVF fertilisation rates, were selected and the men re‐called to deliver an additional semen sample. Proteins involved in gamete membrane fusion on spermatozoa (IZUMO1, SPESP1 and Syncytin‐1) as well as O‐glycosylation patterns (Tn and GALNT3), were investigated by immunofluorescence. The DNA fragmentation index, acrosomal integrity and viability of spermatozoa were determined by flow and image cytometry.

**Results:**

No significant changes in the expression of GALNT3, Tn and Syncytin‐1 were observed between the TFF and control groups. The fraction of spermatozoa expressing SPESP1, the median IZUMO1 staining intensity, and the percentage of viable acrosome‐intact spermatozoa were significantly lower in the TFF group compared to controls. Furthermore, following progesterone‐induced acrosomal exocytosis, a significant difference in the fraction of spermatozoa expressing SPESP1 and the median IZUMO1 staining intensity were observed between the control and TFF group.

**Discussion and conclusion:**

Our results indicate that acrosomal exocytosis, IZUMO1 and SPESP1 expression in spermatozoa could play a crucial role in achieving fertilisation during IVF. However, the size of our cohort was quite small, and our results need to be validated with quantitative methods in larger cohorts.

## 1 INTRODUCTION

With a growing incidence of infertility, more and more couples seek assistance from fertility clinics to achieve pregnancy.^1^ At the fertility clinic, several options exist to help infertile couples. In vitro fertilisation (IVF) describes procedures, where the female is stimulated with gonadotropins to increase the number of mature oocytes, which are then collected and mixed with spermatozoa purified in the lab. Usually, IVF is monitored by the presence of extruded polar bodies, the presence of two pronuclei on day 1, cell divisions on day 2, and the embryo may subsequently be transferred back into the female to achieve a viable pregnancy. Several factors should be considered when choosing IVF. One of the most important factors for IVF to be successful is a sufficient number of spermatozoa. If the sperm concentration is too low for IVF, the couple may choose intracytoplasmic sperm injection (ICSI), where a single spermatozoa is injected directly into the mature oocyte. A subset of the couples that are selected for IVF is, however, faced with total fertilisation failure (TFF), where no zygotes develop despite oocytes and semen parameters appear to be normal. Approximately 5%–15% of all infertile couples experience TFF during IVF, which is a very stressful experience for the couples.^2^ IVF significantly reduces the journey of the spermatozoa, as it circumvents the passage through the uterus and the initial part of the fallopian tubes. However, the spermatozoa must still be able to locate the egg and penetrate the zona pellucida before gamete fusion can occur. For couples persistently experiencing TFF during IVF, it is hence likely that either zona penetration or membrane fusion is impaired.

Membrane fusion between the spermatozoa and the oocyte is a prerequisite for fertilisation to occur. Knowledge of the mechanisms controlling membrane fusion of gametes is still limited, and the proteins initiating sperm–egg fusion remain unknown.^3^ In general, membrane fusion is thought to be initiated at the equatorial segment in spermatozoa and is only possible after acrosomal exocytosis.^4^ Membrane fusion involves the recognition and binding between the two gametes followed by the fusion and merging of the two lipid bilayers, resulting in mixing of the cytoplasm and cellular contents. The membrane protein IZUMO1 on spermatozoa and its oocyte counterpart, JUNO, were the first pair of membrane proteins described to allow the sperm–oocyte interaction and facilitate membrane fusion.^5^ Very recently, FIMP, SOF1, TMEM95 and SPACA6 were also found important for membrane fusion in mice.^6^, ^7^, ^8^ Additional proteins have also been suggested to be implicated in gamete adhesion, including CRISP1 and 2, SPESP1, Integrins, and the ADAM protein family (reviewed in Cuasnicú et al.^4^). Most of these proteins are found on the equatorial segment of spermatozoa following the acrosome reaction, and in general membrane fusion is thought to occur at the equatorial segment of spermatozoa. Our group recently found Syncytin‐1 (ERVW‐1) to locate to the equatorial segment of spermatozoa, and its receptor ASCT2, an amino acid transporter, to be expressed by both spermatozoa and oocytes.^9^ Syncytin is an endogenous retroviral envelope protein originating from the HERV‐W virus family and is involved in the cell–cell fusion in the placenta, bones, muscle and immune system.^10^ Another protein located in the equatorial segment is the polypeptide *N*‐acetylgalactosaminyltransferase 3, also known as GalNac‐T3. *N*‐acetylgalactosaminyltransferases are typically redundantly expressed in different tissues, but GalNAc‐T3 is currently the only known *N*‐acetylgalactosaminyltransferase expressed in spermatozoa.^11^, ^12^, ^13^ GalNAc‐T3 knockout mice produce very few mature spermatozoa with a dysfunctional acrosome and abnormal morphology.^14^ Expression of GalNAc‐T3 in the equatorial segment correlates with semen quality parameters,^13^ and the unique presence of immature O‐glycosylation (Tn) on spermatozoa is likely also involved in membrane fusion events. However, our knowledge of the molecular components of gamete recognition and membrane fusion, their functional interaction and potential species differences is still limited.

Here, we question whether altered expression of membrane fusion proteins IZUMO1, SPESP1 and Syncytin‐1 as well as O‐glycosylation patterns (Tn and GALNT3) on spermatozoa could be related to TFF during IVF. Men in couples experiencing TFF during IVF were recalled to provide an additional semen sample and the spermatozoa investigated for expression of proteins involved in membrane fusion before and after induction of acrosomal exocytosis.

## 2 METHODS

### 2.1 Ethical approval

The retrospective study was conducted at the Fertility Clinic of Rigshospitalet, University Hospital of Copenhagen, Denmark. Patients were recruited between the years 2017–2018 and gave signed informed consent prior to enrolment. The study protocol was approved by the Research Ethics Committee of the Capital Region of Denmark (H‐17012149) and all research was performed in accordance with relevant guidelines and regulations.

### 2.2 Patients

From our database on infertile couples (not able to conceive after at least one year of unprotected sex) receiving fertility treatment, we selected a group consisting of 17 infertile couples that experienced TFF following IVF treatment. The control group consisted of 16 matched couples without TFF after IVF.

In the selection of the TFF group, the inclusion criteria were as follows: (i) ≥ 4 oocytes retrieved for IVF treatment; (ii) sperm concentration ≥ 15 × 10^6^ cells/ml; and (iii) successful fertilisation in a subsequent ICSI treatment. The exclusion criteria were as follows: (i) couples with known female or male causes of infertility affecting oocyte or semen quality, and (ii) couples with donor cycles.

In the selection of the control group, the inclusion criteria were as follows: (i) ≥ 4 oocytes retrieved for IVF treatment; (ii) sperm concentration ≥ 15 × 10^6^ cells/ml; and (iii) a fertilisation rate of 60% or more. They were matched according to the female age in the TFF group (±2 years) and were selected among patients receiving treatment two months prior to or after the couples experiencing TFF. Controls were also selected to match the oocyte and sperm counts of the TFF group, but this was not always possible.

Men in the TFF and control groups were re‐called on average 4 years after IVF, to deliver and additional semen sample, which was investigated for expression of membrane fusion proteins (see below).

### 2.3 Semen processing

All participants had been instructed to abstain from ejaculation at least 48 h before delivery of their semen sample. All samples were produced by masturbation and collected into clean, wide‐mouthed plastic containers. Samples were allowed to liquefy and mixed thoroughly before analysis. Semen analysis included the assessment of volume by weighing. Sperm concentration was determined by image cytometry using a NucleoCounter® NC‐3000™ (ChemoMetec A/S, Allerød, Denmark) as described in Egeberg et al. ^15^ Both sperm motility and morphology were assessed according to WHO guidelines with the modifications described by Jørgensen et al.^16^ In brief, sperm motility was evaluated by adding 10 µl semen to a heated (37°C) glass slide, which was immediately examined on a heated (37°C) stage of a microscope and spermatozoa classified as progressive motile or non‐progressive/immotile. For assessment of sperm morphology, semen smears were Papanicolaou stained, and evaluated by two experienced technicians according to “Tygerberg stricter criteria” as reported in Menkveld et al. ^17^ The semen analysis was performed at the Department of Growth and Reproduction, accredited by the European Andrology Academy (EAA) and participating in annual ESHRE quality control programmes. Total progressive motile sperm count (TPMSC) was calculated by multiplying the percentage of progressive motile spermatozoa with the concentration.

Aliquots of raw semen corresponding to 3 × 10^6^ cells were immediately used for the assessment of acrosomal status and viability. Additional raw semen aliquots of 200 µl were frozen at −80°C for sperm chromatin structure assay (SCSA) analysis. The remaining samples were purified by density gradient centrifugation (DGC) as described before,^18^ and either directly used for immunofluorescence (IF) analysis or, capacitated and acrosome induced prior to IF analysis.

Due to the limited number of spermatozoa in each ejaculate, some samples could not be analysed for all parameters. Hence, 4 and 1 men were re‐called twice in the control and TFF group, respectively. The total number of samples analysed in each group, therefore, ranges from 20 to 16 in the control group and 18 to 13 in the TFF group.

### 2.4 Assessment of acrosomal status and viability

The fraction of acrosome‐intact spermatozoa was determined as described in detail before.^19^ Briefly, aliquots of 3 × 10^6^ cells were washed twice in Dulbecco's phosphate‐buffered saline (PBS, ThermoFischer scientific) (500×*g*, 10 min), and the pellet re‐suspended in a staining solution containing (final concentrations): 5 µg/ml fluorescein isothiocyanate conjugated *Pisum Sativum* agglutinin (FITC‐PSA, Sigma–Aldrich, MO, USA), 0.5 µl/ml propidium iodide (PI, ChemoMetec A/S, Allerød, Denmark) and 10 µl/ml Hoechst‐33342 (H342, ChemoMetec A/S, Allerød, Denmark) in PBS. The samples were incubated at 37°C for 30 min on a shaker, followed by mixing with 100 µl of an immobilising solution containing 0.6 M NaHCO_3_ and 0.37% (v/v) formaldehyde in distilled water. The samples were loaded onto two‐chamber NC‐Slide A2™ slides (ChemoMetec) and analysed by image cytometry using a NucleoCounter® NC‐3000™ (ChemoMetec) and a FlexiCyte protocol. The PI intensity was plotted as a function of FITC‐PSA intensity on bi‐exponential scales, and specific quadrant gates, as published before,^19^ were used to identify the fraction of acrosome‐intact viable spermatozoa.

### 2.5 DNA integrity

Spermatozoal DNA integrity was assessed at the Reproductive Medicine Centre, Skåne University Hospital, Malmo, Sweden, as described elsewhere.^20^, ^21^ Briefly, thawed raw semen samples were treated with a buffer with pH 1.2 for 30 s to denature the DNA at the sites of strand breaks. Subsequently, the fluorescent DNA dye acridine orange was added, and samples analysed by flow cytometry. Denatured (single‐stranded) DNA emits red fluorescence, and the intact (double‐stranded) DNA emits green fluorescence. The percentage of red spermatozoa is called the DNA fragmentation index (DFI) and represents the fraction of spermatozoa with denatured DNA. High DNA stainable (HDS) spermatozoa are the fraction of spermatozoa with the most intense green colour and are believed to represent immature spermatozoa.^22^

### 2.6 Purification, capacitation and acrosome reaction

Semen samples were purified using a discontinuous 40%/80% density gradient (PureSperm, Nidacon) enabling motile spermatozoa to be separated from immotile/immature spermatozoa, extraneous cells, and seminal plasma. Cell pellets were re‐suspended in 2 ml 4 mM human tubular fluid (HTF+) medium containing (in mM): 72.8 NaCl, 4.69 KCl, 0.2 MgSO_4_, 0.37 KH_2_PO_4_, 2.04 CaCl_2_, 0.33 sodium pyruvate, 21.4 sodium lactate, 2.78 glucose, 21 HEPES, and 25 NaHCO_3_, adjusted to pH 7.4 with NaOH for 1 h at 37°C with 10% CO_2_ in the air, and the concentration and motility were determined as described above.

For capacitation, the cell concentration was adjusted to 10 × 10^6^ cells/ml in HTF+ medium containing 25 mM NaHCO_3_ and 3 mg/ml (3% [v:v]) human serum albumin (HSA, Irvine Scientific, CA, USA) and incubated for at least 3 h at 37°C:10% CO_2_. After capacitation, the spermatozoa were induced to undergo acrosome reaction by adding 10 µM progesterone (Sigma–Aldrich, MO, USA) and incubated for 30 min at 37°C.

### 2.7 Immunofluorescence

Preparations of cytospins and subsequent IF analysis were performed on aliquots drawn from the motile fraction after density gradient purification and aliquots of the acrosome‐reacted samples. Approximately, 100,000 spermatozoa were spun down on a microscopy slide with a cytospin cytocentrifuge (Shandon Cytospin 4, Thermo Scientific) at 15,000×*g* for 5 min. The slides were left to air‐dry after which the cells were fixated with either 100% ice‐cold acetone/ethanol for 10 min or formalin fixation for 10 min (Table 1), followed by 3 × 5 min wash in tris‐buffered saline (TBS). Formalin‐fixed cells were permeabilised with 0.1% Triton‐X100 for 10 min. Slides were then washed in TBS before incubation with 5% bovine serum albumin (BSA; Fraction V, Roche Diagnostics) for 30 min at room temperature, in a humidified dark chamber. Next, slides were incubated with their respective primary antibodies (Table 1; all validated in independent publications), overnight at 4°C. Exclusion of the primary antibody was used as a negative control. The next day, slides were left at room temperature for 1 h, washed in TBS to remove excess primary antibody and incubated with secondary antibody for 1 h at room temperature. The slides were washed in TBS and briefly stained with 4,6‐diamidino‐2‐phenylindole (DAPI 1:600; Sigma) for nuclear staining followed by a wash in TBS. Lastly, the slides were rinsed in double‐distilled water, the excess liquid wiped away and the slides mounted in ProLong™ Gold antifade reagent (Invitrogen, Nærum, Denmark), and covered with a coverslip.

**TABLE 1:** List of primary and secondary antibodies used in the study

**Primary antibodies**	**Type**	**Host**	**Isotype**	**Fixative**	**Dilution**	**Source**
GalNAc‐T3^13^	Primary	Mouse monoclonal	IgG	100% ice‐cold acetone	Undil.	mAb UH5 (clone 2D10). A kind gift from Dr Ulla Mandel, UCPH.
IZUMO1^8^	Primary	Rabbit polyclonal	IgG	Formalin 10%	1:200	Ab211623. Lot. GR273252‐5 Abcam
SPESP1^33^	Primary	Rabbit polyclonal	IgG	100% ice‐cold acetone	1:200	HPA045936. Lot. R42149 Sigma–Aldrich
Syncytin‐1^9^	Primary	Rabbit polyclonal	IgG	Formalin 10%	1:50	sc‐50369 (H‐280) Lot. J2612 Santa Cruz Biotechnology
Tn (mAb 5F4)^13^	Primary	Mouse monoclonal	IgM	100% ice‐cold acetone	Undil.	A kind gift from Dr Ulla Mandel, UCPH.
Donkey‐anti‐mouse Alexa Fluor ®488	Secondary	Donkey	A‐21202	NA	1:600	Thermo Fischer Scientific
Donkey‐anti‐rabbit Alexa Fluor ®488	Secondary	Donkey	A‐21206	NA	1:600	Thermo Fischer Scientific

### 2.8 Image acquisition and segmentation

Slides were visualised with an Olympus BX‐61 microscope, and images were captured using Cell Sens Dimensions V1.6 software (Olympus Ltd.). It was not possible to use standardised image acquisition properties for all antibodies and experiments and, hence, we sought to acquire images best representing the staining of each experiment. Further image processing was performed with FIJI ImageJ 1.49 M by using a mask quantification protocol, as outlined before.^23^ Briefly, DAPI (blue) and FITC (green) channels were split and converted to grayscale, followed by image background correction. Subsequently, all structures of interest (spermatozoa heads only) were segmented on the DAPI channel and designated as regions of interest (ROIs). These ROIs were then merged onto the green channel showing the FITC‐labelled target molecule, and thus the fluorescence intensity of GALNT3, Tn, IZUMO1, SPEPS1 and Syncytin‐1 in each spermatozoa head only. Cells were additionally sorted by area, disregarding very small and very large cells, to isolate morphologically normal spermatozoa for the analysis. From the ROIs, we measured the mean intensity in the FITC‐channel and used this as a measure of the mean intensity per spermatozoa. The mean intensity per spermatozoa hence represents a distribution of staining intensities describing biological variation observed in the entire ejaculate and we used the median of this distribution to describe the staining intensity of each sample. For the calculation of the percentage of positive cells, those with a detected intensity lower than the established autofluorescence were considered negative.

### 2.9 Statistical analysis

Statistical analysis was done using the software RStudio 1.2.5001. The nonparametric Wilcoxon rank sum test was used to compare parameters between the TFF and control groups as well as between the mean fluorescence intensity of populations of spermatozoa before and after induction of acrosomal exocytosis. All plots were produced with the *ggpubr* package in R. *p*‐values below 0.05 were considered significant.

## 3 RESULTS

### 3.1 General characteristics of total fertilisation failure and control groups

From our database on couples receiving fertility treatment, a total of 33 couples were selected for the study, 17 of which experienced TFF (group) during IVF treatment and 16 that did not (control group).

There were no significant differences between the primary cause of infertility, albeit more male‐ and less female‐factor couples were observed in the TFF group (Table 2). Also, no significant difference was observed in female or male age, sperm concentration, percentage progressive motile spermatozoa or in the total progressively motile sperm count (TPMSC; Table 3). However, parameters measured after DGC showed significant differences between control and TFF patients (Table 3). In the control group, the mean post‐purification TPMSC (40.6 mill/ml) and mean TPMSC recovery rate (118.3%) were significantly higher than in the TFF group (24.1 mill/ml (*p* < 0.05) and 64.2% (*p* < 0.01), respectively, Table 3).

**TABLE 2:** Indications for IVF in control and TFF groups

**IVF indication**	**Control (*n* = 16)**	**TFF (*n* = 17)**	***p‐*value**
Unexplained infertility	7 (44%)	8 (47%)	NS
Male factor	1 (6%)	5 (29%)	NS
Female factor	5 (31%)	2 (12%)	NS
More than one reason	3 (19%)	2 (12%)	NS

*Notes*: Data are expressed as *n* (%); *p*‐values are based on Fisher's exact test.

Abbreviations: IVF, in vitro fertilisation; TFF, total fertilisation failure.

**TABLE 3:** Baseline characteristics of control and TFF groups at time of fertility treatment. Data are expressed as mean ± SD, median (range)

**Characteristic**	**Control (*n* = 16)**	**TFF (*n* = 17)**	***p*‐value**
Female age (years)	33.9 ± 2.6	33.9 ± 3.6	NS
Male age (years)	36.4 ± 4.2	36.8 ± 4.2	NS
Sperm concentration (mill/ml)	67.9 ± 38 70 (20–170)	76.8 ± 57.4 60 (20–280)	NS
Progressive motile spermatozoa (%)	47.3 ± 18.5 47.7 (6.7–75)	47.9 ± 18.7 50 (16.7–80)	NS
TPMSC (mill)	93.3 ± 76.3 60 (15–300)	102.5 ± 75.7 ^a^ 87.5 (3.2–250)	NS
Post‐wash TPMSC (mill)	40.6 ± 25.3 40 (3–90)	24.1 ± 21.4 18 (2.8–90)	< 0.05
TPMSC recovery rate (%)	118.3 ± 64.9 100 (9.4–225)	64.2 ± 41 53 (4.9–187.5)	< 0.01

*Note*: ^a^ *n* = 16.

Abbreviations: NS, not significant; TFF, total fertilisation failure; TPMSC, total progressive motile sperm count.

Men included in the two groups were re‐called to provide new semen samples for the study. Because progressive motility was not measured following DGC, the post‐purification sperm concentration, and total sperm count (TSC) were compared between the groups, to see if observations regarding post‐purification parameters would replicate (Table 4). Additionally, the acrosomal status, spermatozoa morphology, immunofluorescent staining and the SCSA were performed on the re‐call samples. Samples at recall showed a significantly better progressive motility (control group *p *= 0.0001; TFF group *p *= 0.035) compared to the samples used at fertility treatment. Also, the sperm concentration tended to improve, albeit this was insignificant.

**TABLE 4:** Semen parameters of control and TFF groups at recall

**Sperm parameter**	**Control**	**TFF**	***p*‐value**
Sperm concentration (mill/ml)	91.1 ± 49.8^a^ 90.5 (6.7–186.5)	78.6 ± 60.9^b^ 61.7 (2.6–199.4)	NS
Progressive motile spermatozoa (%)	70.3 ± 10.3^b^ 70.5 (54.5–88.5)	61.4 ± 17^b^ 65.5 (20.5–80.5)	NS
TPMSC (mill)	61.3 ± 40.4^b^ 57.9 (5.6–165.1)	49.2 ± 38.2^c^ 45.1 (0.5–148.6)	NS
Post‐wash concentration (mill/ml)	19.6 ± 20.6^d^ 10.4 (3–74.9)	7.7 ± 5.8^b^ 5.2 (2.4–21)	< 0.05
TSC recovery rate (%)	12.2 ± 7.2^a^ 10.1 (0.53–28.9)	8.7 ± 11.1^b^ 5 (1.4–51.5)	< 0.01
Morphology, normal (%)	11.5 ± 4.7^e^ 9.5 (6–21.5)	8.9 ± 3.2g^f^ 8.8 (4.5–14)	NS
DFI (%)	16.2 ± 7^e^ 15.5 (9–33)	18.4 ± 11.7^g^ 13.3 (7–48)	NS
HDS (%)	14.1 ± 7.8^e^ 12 (2–33)	18.2 ± 11.8^g^ 12.5 (8–48)	NS

*Notes*: Data are expressed as mean ± SD, median (range).

^a^ *n* = 20.

^b^ *n* = 17.

^c^ *n* = 18.

^d^ *n* = 19.

^e^ *n* = 16.

^f^ *n* = 13.

^g^ *n* = 15.

Abbreviations: DFI, DNA fragmentation index; HDS, high DNA stainable; NS, not significant; TFF, total fertilisation failure; TPMSC, total progressive motile sperm count; TSC, total sperm count. .

At re‐call, there were no significant differences between the two groups in sperm concentration, percentage of motile progressive spermatozoa, TPMSC, percentage of morphologically normal spermatozoa, DFI or HDS (Table 4). However, the mean post‐purification concentration and mean TSC recovery rate were significantly higher in the control group (19.6 mill/ml and 12.2%, respectively), compared with the TFF group (7.7 mill/ml (*p* < 0.05) and 8.7% (*p* < 0.01), respectively; Table 4).

These results mirror those from the time of fertility treatment, suggesting a correlation between post‐purification recovery rates and fertilisation outcomes.

### 3.2 Localisation of GALNT3, IZUMO1, SPESP1 and Syncytin‐1 in spermatozoa

TFF could be caused by differences in membrane fusion proteins. To test this hypothesis, spermatozoa from men in the control and TFF groups were stained for GALNT3, Tn, IZUMO1, SPESP1 and Syncytin‐1.

**FIGURE 1:**
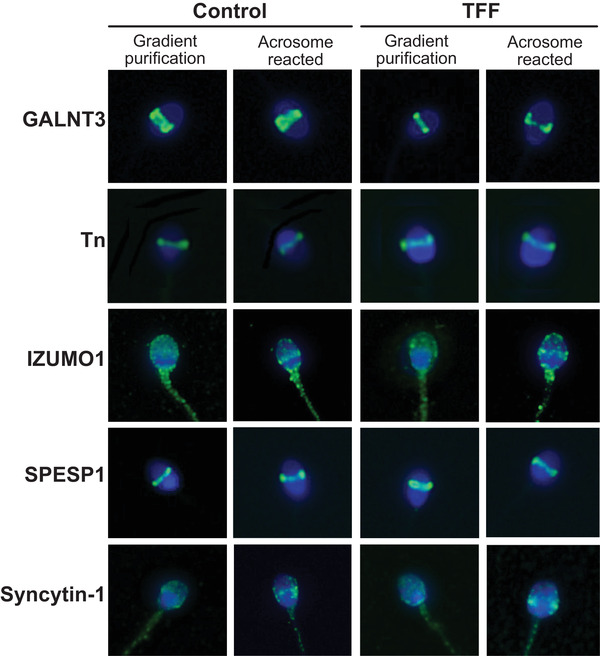
The localisation of GALNT3, Tn, IZUMO1, SPESP1 and Syncytin‐1 in density gradient centrifugation (DGC) and acrosomal exocytosis (AR) human spermatozoa from the control and total fertilisation failure (TFF) groups. Representative immunofluorescence images of expression in individual spermatozoa labelled with anti‐GALNT3, anti‐Tn, anti‐IZUMO1, anti‐SPESP1 and anti‐Syncytin‐1 antibodies and fluorescein isothiocyanate (FITC)‐conjugated secondary antibodies (green). Nuclei (DNA) were counterstained with diamidino‐2‐phenylindole (DAPI, blue). No apparent visual differences were observed between staining patterns in the control and TFF groups, but a slight tendency for staining to gather in the equatorial segment following induction of the acrosome reaction was observed with IZUMO1 and Syncytin‐1

Figure 1 shows the representative localisation patterns of GALNT3, Tn, IZUMO1, SPESP1 and Syncytin‐1, in spermatozoa purified by DGC and spermatozoa with progesterone‐induced acrosomal exocytosis (AR) from control and TFF patients. No apparent visual differences were found between the control and TFF groups. Both GALNT3 and SPESP1 showed similar staining localised to the equatorial segment of the spermatozoa head (Figure 1). Also, staining of the immature *O*‐glycan Tn was also detected in the equatorial segment of DGC and AR spermatozoa from both control and TFF patients (Figure 1). The localisation of IZUMO1 and Syncytin‐1 was noticeably different from that of GALNT3, Tn and SPESP1 (Figure 1) and in the DGC faction of both control and TFF groups, IZUMO1 and Syncytin‐1 localised in the acrosomal cap region, with visible diffusion to the entire head (Figure 1). Slight staining of the midpiece and tail were also noted in all spermatozoa. Following progesterone‐induction of the acrosome reaction, both IZUMO1 and Syncytin‐1 showed a tendency to gather at the equatorial segment (Figure 1). In both groups, a subset of cells did not display this tendency and retained the entire head distribution fluorescence pattern.

These results suggest a translocation of IZUMO1 and Syncytin‐1 to the equatorial segment during the acrosome reaction.

### 3.3 Expression and intensity of GALNT3, Tn, IZUMO1, SPESP1 and Syncytin‐1 in DGC human spermatozoa

The intensity of the staining patterns on all images was analysed using the ImageJ software, as described above (see Section 2). In total, heads of 213,799 spermatozoa were analysed and the mean fluorescence intensity in each head determined. Subsequently, the median fluorescence intensity per sample was determined.

**FIGURE 2:**
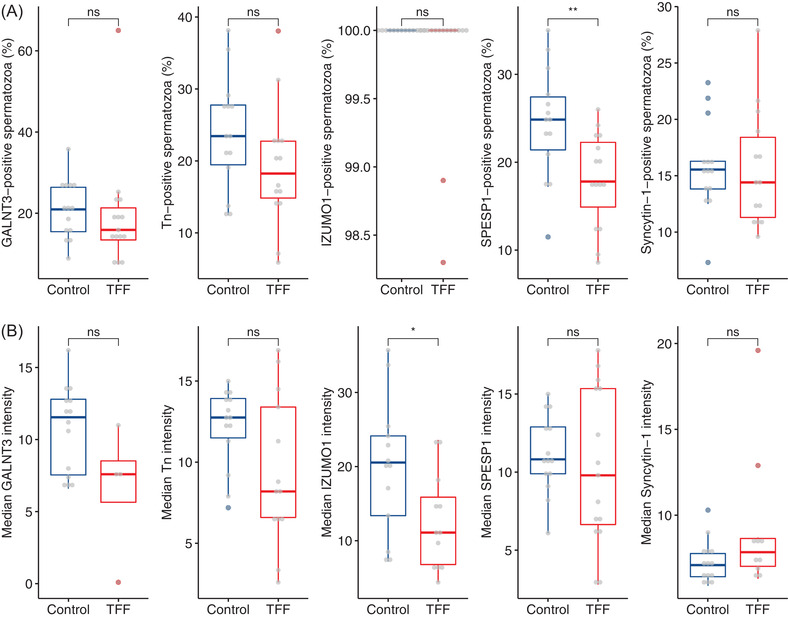
Evaluation of expression and relative quantification of GALNT3, Tn, IZUMO1, SPESP1 and Syncytin‐1 in density gradient centrifugation (DGC) spermatozoa from control and total fertilisation failure (TFF) groups. (A) Percentage of GALNT3, Tn, IZUMO1, SPESP1 and Syncytin‐1 expressing cells in control and TFF groups. (B) Median fluorescence intensity of ejaculates stained for GALNT3 (*n* = 29), Tn (*n* = 27), IZUMO1 (*n* = 28), SPESP1 (*n* = 29) and Syncytin‐1 (*n* = 28) in control and TFF groups. The box plots depict the interquartile ranges, with whiskers at 1.5 times the interquartile range. * *p *< 0.05, ** *p *< 0.01

Figure 2 shows the percentage of DGC spermatozoa positive for GALNT3, Tn, IZUMO1, SPESP1 and Syncytin‐1, as well as the median fluorescence intensities, from the control and TFF groups. The fraction of spermatozoa expressing SPESP1 was significantly higher in the control group compared with TFF, with a median of 24.8% versus 17.8% positive cells, respectively (*p *= 0.008) (Figure 2A). Although not statistically significant, the number of spermatozoa expressing GALNT3, Tn and Syncytin‐1 was also higher in the control group compared with TFF (median of 21% and 15.9%; 23.4% and 18.2%; 15.5% and 14.4%, respectively; Figure 2A). Contrarily, the percentage of IZUMO1 expressing cells was 100% in both control and TFF groups (Figure 2A).

The median fluorescence intensity of IZUMO1 was significantly higher in the control group compared with TFF (20.6 vs. 11.1; *p* = 0.041, Figure 2B). The median intensities of GALNT3, Tn, SPESP1 and Syncytin‐1 were not significantly different between the control and the TFF group (Figure 2B). In general, the median intensity and the fraction of positive spermatozoa showed similar directions in the regulation between the groups.

These results highlight a significantly lower number of spermatozoa positive for SPESP1 and a significant lower intensity of IZUMO1 expression in spermatozoa from men in couples with TFF.

### 3.4 Acrosomal status assessment in ejaculates

**FIGURE 3:**
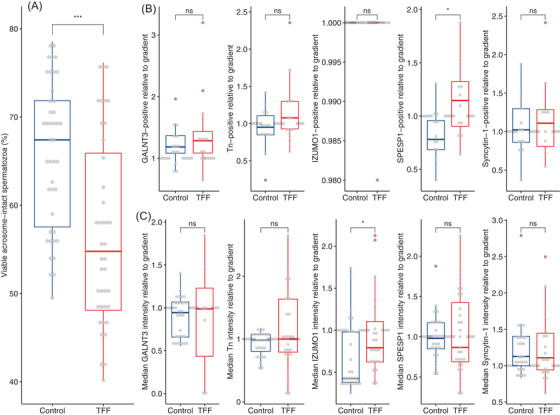
Acrosomal status and the effect of inducing acrosomal exocytosis (AR) on expression and relative intensity of GALNT3, IZUMO1, SPESP1 and Syncytin‐1. (A) Assessment of acrosomal status in washed ejaculates. ***p < 0.001. (B) Change in the number of spermatozoa positive for GALNT3, Tn, IZUMO1, SPESP1 and Syncytin‐1 in acrosome reacted spermatozoa from control and total fertilisation failure (TFF) groups, relative to density gradient centrifugation (DGC) spermatozoa (Figure 2, Figure S1). *p < 0.05. (C) Change in the median fluorescence intensity of acrosome reacted ejaculates stained for GALNT3, Tn, IZUMO1, SPESP1 and Syncytin‐1 in control and TFF groups, relative to DGC spermatozoa (Figure 2, Figure S2). The box plots depict the interquartile ranges, with whiskers at 1.5 times the interquartile range. *p < 0.05

Because only spermatozoa with an intact acrosome can successfully fertilise an oocyte, the acrosomal status of raw ejaculates from both control and TFF patients was assessed by image cytometry. Figure 3A shows the fraction of viable acrosome‐intact spermatozoa from both groups. The percentage of viable acrosome‐intact spermatozoa was significantly higher in the control group compared with TFF (median 67.4% and 54.8%, respectively; *p* < 0.001, Figure 3A).

These results indicate that the presence of an intact acrosome correlates with TFF and highlights the importance of an intact acrosome for successful IVF.

### 3.5 Effect of inducing the acrosome reaction on expression and intensity of GALNT3, IZUMO1, SPESP1 and Syncytin‐1

Following DGC, progesterone‐mediated acrosomal exocytosis was performed in an aliquot of the sample, and the expression and intensity of the target molecules were assessed by the same protocol as described above.

Inducing the acrosome reaction had no significant effect on the fraction of spermatozoa expressing GALNT3, Tn, IZUMO1 or Syncytin‐1 in either the control or TFF group (Figure 3B, Figure S1). However, as expected,^13^ the percentage of GALNT3 positive cells increased (but not significantly) in the AR population compared with DGC in both control and TFF patients (1.2‐fold and 1.3‐fold, respectively, Figure 3B, Figure S1). Interestingly, inducing the acrosome reaction led to a 0.2‐fold decrease in SPESP1 expression in the control group (Figure 3B) but a 1.2‐fold increase in the TFF group (*p *= 0.016; Figure 3B, Figure S1).

The median fluorescence intensity of target molecules GALNT3, Tn and SPESP1 were not affected by the acrosome reaction in both control and TFF patients (Figure 3C, Figure S2). IZUMO1 fluorescence intensity showed a significant (*p *= 0.003) decrease (0.5‐fold) and Syncytin‐1 a significant (*p *= 0.0005) increase (1.4‐fold) in the control group, which was not observed in the TFF group (Figure S2). However, only for IZUMO1 the difference in staining intensity after acrosomal exocytosis was significantly (*p *= 0.014) different between the control and TFF group (Figure 3C).

Taken together, these results indicate that both the number of SPESP1 positive spermatozoa and the IZUMO1 expression level decrease after acrosomal exocytosis in the control group, but not in the TFF group.

## 4 DISCUSSION

To our knowledge, our study represents one of the first cohort‐based studies to relate TFF during IVF to the expression of membrane fusion proteins. Although we did not find visually altered expression patterns of proteins involved in membrane fusion, our results indicate that changes in the number of SPESP1‐positive and the IZUMO1 expression levels exist in spermatozoa from men experiencing TFF. In addition, we found evidence suggesting that spermatozoa from men experiencing TFF have impaired acrosomal integrity and that SPESP1 and IZUMO1 may be unable to respond to acrosomal exocytosis. After induction of the acrosome reaction only SPESP1 showed significant different number of spermatozoa being positive and IZUMO1 showed significant changes in median intensity. Hence, several of our results indicate that SPESP1 and IZUMO1 could play a crucial role in achieving fertilisation during IVF. Studies of SPESP1 in rodents also support this. Application of an anti‐SPESP1 antibody inhibited sperm–egg fusion in the human sperm–hamster egg system,^24^ and *Spesp1* knockout male mice showed reduced fertility and inhibition of sperm–oocyte fusion.^25^ Furthermore, the equatorial expression of ADAM family proteins and MN9 was disrupted in *Spesp1* knockout male mice indicating that SPESP1 is a central player in gamete membrane fusion.^25^ Obviously, IZUMO1 is also a central player in membrane fusion and genetic studies have indicated that missense variants in *IZUMO1R* (encoding JUNO) were more frequent among women experiencing TFF.^26^ Furthermore, inhibiting spermatozoal IZUMO1‐binding to JUNO on oocytes, by addition of an anti‐JUNO antibody, completely blocked gamete fusion.^27^

It can be questioned whether changes in median intensity represent a valuable read‐out since measuring IF is not a direct quantitative method and that we were unable to standardise our measurements. The advantage is, however, that the staining pattern of many cells can be evaluated unbiased and without loss of spatial information. Recent studies have also reported that quantification of IF by segmentation in ImageJ/Fiji represent a reliable method for quantification.^23^ At least in human spermatozoa, induction of acrosomal exocytosis with progesterone is not complete (only ∼20% are expected to react) and a substantial proportion of the population will retain an intact acrosome.^19^ This could explain why we visually do not observe more remodelling of equatorial proteins after induction of acrosomal exocytosis, and that effects are only observed when a large population of spermatozoa is evaluated, for example, by analysing the mean intensity.

Limitations of our study include a small cohort size and that only a selected subset of proteins described to be involved in membrane fusion was tested. Even though the fertility clinic at Rigshospitalet is the biggest public fertility clinic in Denmark, we were only able to recruit 17 couples with TFF during IVF. Ideally, more couples from different clinics should be included, but the number should also be balanced with the increase in workload of screening each sample for membrane fusion proteins. The small cohort size also limited our options in selecting on other parameters like sperm concentration. At the same time, some of the men had a rather low sperm concentration, albeit within the normal range (defined by WHO), which again limited our options to stain for additional membrane fusion proteins, like TSSK6, SPACA6, and BPI,^4^ our options to using more sensitive methods like flow‐cytometry and our options to validate our findings by, for example, Western blot. We tried to optimise antibodies against CRISP1 and 2, but we were unable to get consistent staining patterns with these antibodies, maybe due to their ability to oligomerise.^28^ Finally, we did not consider membrane fusion proteins on the oocyte. It is known, for example, that CD9 on the oocyte is essential for membrane fusion.^29^, ^30^, ^31^ It is, however, challenging to obtain unfertilised eggs from a cohort of females experiencing TFF. Finally, other sperm parameters could be involved in TFF, including inability to hyperactivate, which we did not investigate, and we were also not able to analyse specific sub‐groups like male versus female factor infertility.

Future studies should include even larger cohorts of men experiencing TFF and include additional proteins implicated in membrane fusion. After this study was initiated, additional membrane fusion candidates have been identified, including FIMP, SOF1, and TMEM95, and it would be very interesting to investigate differences in the expression of such candidates.^6^, ^7^ Clinically, couples experiencing TFF during IVF could obviously be helped by the use of ICSI but other options, like addition oocyte activating factors,^32^ could possible also be used to promote gamete fusion.

## 5 CONCLUSION

We found significant changes in the number of SPESP1 positive spermatozoa and IZUMO1 expression in spermatozoa from men experiencing TFF. Also, men experiencing TFF had impaired acrosomal integrity, and both SPESP1 and IZUMO1 appeared inert to induction of acrosomal exocytosis. Our results indicate that SPESP1 and IZUMO1 could play a crucial role in achieving fertilisation during IVF.

## AUTHORS CONTRIBUTION

Conceptualization: Marie Berg Nygaard, Søren Ziebe, Morten R. Petersen, Kristian Almstrup; experimental procedures and analysis: Simona Ioana Enoiu, Marie Berg Nygaard, Mona Bungum, Kristian Almstrup; manuscript drafting: Simona Ioana Enoiu, Kristian Almstrup; manuscript review and editing: all authors. All authors have read and agreed to the published version of the manuscript.

## CONFLICT OF INTEREST

The authors declare that there is no conflict of interest that could be perceived as prejudicing the impartiality of the research reported.

## Supporting information

Supporting Information
