# Supplementary material for: Expression of membrane fusion proteins in spermatozoa and total fertilisation failure during in vitro fertilisation
Source: Andrology. 2022 Jul 1;10(7):1317–27. doi: 10.1111/andr.13215 (PMC9540887; doi:10.1111/andr.13215)
Supplement: Supplementary file 1 — Supporting Information [file ANDR-10-1317-s001.docx]

# Supplementary Figures

#

**Supplementary figure S1.** Boxplots showing change in the number of spermatozoa positive for GALNT3, IZUMO1, SPESP1, Syncytin-1 and Tn in Control and TFF groups relative to DGC spermatozoa and plotted for both the reference DGC spermatozoa (GRAD) and acrosome reacted (AR) populations.

**Supplementary figure S2.** Boxplots showing change in the median fluorescence intensity for GALNT3, IZUMO1, SPESP1, Syncytin-1 and Tn in Control and TFF groups relative to DGC spermatozoa and plotted for both the reference DGC spermatozoa (GRAD) and acrosome reacted (AR) populations. ** *p*<0.01, *** *p*<0.001.
